# Book Review

**Published:** 1901-12

**Authors:** 


**Motherhood.** By Charles J. Glasson, M.D. Pp. 91. London: John Bale, Sons & Danielsson, Ltd. 1901.—From the author's preface we learn that "this little handbook contains valuable advice to those young girls who are just about to get married, or have entered upon that state as young wives. Advice to mothers in regard to their daughters. Advice to schoolmistresses and others having the charge of young girls in regard to their training. Natural and artificial feeding of infants. It is intended as a help and a guide to those who have no knowledge of Motherhood, and contains nothing that will offend the modesty of any woman." We think the author has carried out his intentions with much common sense. It would save many a mother much difficulty if the statement (page 25) could be looked upon as infallible. He writes: "According as a woman is sparing or generous of fluid, sweet and starchy foods, so the size of her baby will be—small, if sparing; large, if generous. The happy medium is the best."

**Our Baby: for Mothers and Nurses.** By Mrs. Langton Hewer. Seventh Edition. Pp. viii., 154. Bristol: John Wright & Co. 1901.—There is no better guide, as far as we know, for the management and bringing-up of infants.

**Hygiene and Public Health.** By Louis Parkes, M.D., and Henry Kenwood, M.B. Pp. xix., 732. London: H. K. Lewis. 1901.—When a book reaches its fifth edition, it has already proved that it satisfactorily meets a very widespread want; but with each successive edition it becomes increasingly difficult to incorporate new information without destroying that continuity of design, the value of which in imparting knowledge is perhaps, not always fully appreciated. The authors of the work under notice have done wisely in avoiding this difficulty by recasting the whole. The result is an exceedingly able summary of the widely-varied matter that comes within the ken of the medical officer. The description of practical laboratory operations is advisedly left in great measure to special treatises intended to be used test-tube in hand; but ample information is given as to the conclusions to be drawn from analytical data. On the whole it will be difficult to find a more concise, yet reliable, account of points to be considered in the maintenance of the physical well-being of centres of population.

**Dental Surgery.** By Henry Sewill. Fourth Edition. Edited by W. J. England and J. Sefton Sewill. Pp. x., 622. London: Baillière, Tindall, & Cox. 1901.—This new edition of a well-known work is worthy of very high praise indeed. It is as good and useful a text-book as we know. It has been thoroughly re-written and brought up to date. The illustrations are very excellent. On the whole, we consider it fulfils in every respect the aims of its author.

**Manual of Midwifery.** By W. E. Fothergill, M.D. Second Edition. Pp. xviii., 506. Edinburgh: William F. Clay. 1900. —The appearance of the second edition is sufficient evidence of the popularity of this work. In a previous review, published in the March number of this *Journal* for 1897, we pointed out certain places in which the portions of the subject treated required elucidation and addition, and certain statements to which we took exception. We do not find that any alteration has been made in this edition, and therefore repeat our previous criticism. The best parts of the book are the sections on the development of the ovum and placenta, and that on the mechanism of labour and the indications for, and methods of use of, the forceps. These give most complete and accurate descriptions of the processes mentioned. One or two very interesting observations are recorded, notably that by Milne Murray on the effect of hot and cold water respectively on the pelvic vessels, which gives most convincing evidence of the advantage of the hot intra-uterine or vaginal douche for securing uterine contraction. This is found under an excellent account of the *rationale* of the various methods of treating *post-partum* hemorrhage. Another interesting observation recorded is that by Milne Murray on the effect of pressure on the diameters of the fœtal head, showing how little those in the horizontal plane of the occipito-frontal diameter are affected. The author, in treating of the puerperium, speaks of vaginal douching with permanganate of potash as a cleanly and comfortable practice. We demur to the first statement entirely. We do not consider permanganate of potash, *as generally used*, either an efficient antiseptic or even that it can be depended on to render water sterile. We know of one fatal case of septicæmia (infection with streptococcus erysipelatosus) traced to its use. Douching after labour is only safe if sterility of fluid, instruments, and operator's hands can be *insured*; if not, it is better omitted altogether. The remarks on the treatment of the nursing infant might well have been amplified; so far as they go they are excellent. We can recommend the book as a whole as a readable and accurate work.

**Outlines of the Diseases of Women.** By John Phillips, M.D. Third Edition. Pp. xvi., 280. London: Charles Griffin & Company, Limited. 1901.—It is four years since the last edition of this book appeared, and although the general arrangement of the book is the same as in former editions, the rapid advance of gynæcological science has made it necessary to revise some portions of the work, especially the chapters on uterine fibroids and ectopic gestation. With regard to the treatment of fibromyomata of the uterus, Dr. Phillips says that Apostoli's treatment is still on its trial, although in the majority of cases it is quite useless. Discussing the various operative methods of dealing with these tumours, he still advocates removal of the ovaries in certain cases, and looks upon hysterectomy as the last resource. After describing the intra- and extra-peritoneal methods of dealing with the stump, he says there is a growing tendency in favour of the former method. The author thinks that the majority, if not all, cases of extra-uterine pregnancy are primarily tubal, and evidently does not consider the recently recorded cases of primary ovarian pregnancy as convincing. The relative value of supra-vaginal amputation of the cervix and total extirpation of the uterus for cancer is at present, he says, not yet decided, although most authorities consider the former to be a useless proceeding. A very useful chapter has been added on the bacteriology of the genital tract. The book is essentially practical in its nature, and will be found of great value to the student and young practitioner.

**Materia Medica and Therapeutics, with Special Reference to the Clinical Application of Drugs.** By John V. Shoemaker, M.D., LL.D. Fifth Edition. Pp. vii., 766. Philadelphia: F. A. Davis Company. 1900.—Owing to the rapid multiplication of new remedies, especially in America, the author of this standard treatise is issuing it in two forms. The present publication is the *Students' Edition*, and is confined to the consideration of the drugs in the British and United States Pharmacopœias and some of their chemical derivatives. Even with this restriction it extends to 766 pages of close print, and embraces a very large number of drugs, many of which are little known in this country. What the Physicians' or enlarged edition will extend to we hardly like to imagine. One of the most valuable features of the book is the vast number of prescriptions and formulæ which are scattered through its pages. Thus, under cod-liver oil for instance, a dozen methods of making emulsions or special preparations of the oil are given, and an even greater number of formulæ are supplied in the chapter on bromides. The author, as a dermatologist, gives considerable prominence to the employment of various drugs in skin diseases, with copious suggestions as to the choice and combination of remedies. Other departments are, however, by no means neglected; and we observe numerous references to the most recent work by European and American writers. There is one difficulty against the use of the book in this country, from the fact that the difference in strength of British and American preparations is apt to be overlooked. Still, with this caution beforehand, the reader cannot help finding the work of constant use. The early chapters on prescribing are interesting, but too cursory. Thus, on page 47 the hydrostatic douche or fountain enema is mentioned, but no warning is given as to the dangers of too great pressure if the column of fluid is unduly high, or any account of the advantages of the method. The common rubber enema is described in a singularly clumsy sentence which contains one of the few Americanisms in the book: "It is easily operated and can be used as a self-injecting apparatus." There seem to be curious slips in the Latin phrases supplied as aids in writing prescriptions: "Omnis horis=every hour," "omni bihoris=every two hours," "pro ratione ætas=according to age," "more dictu=in the manner written," "macero=to macerate," "infrico=to rub in," "diebus tertius =every third day." This is the more remarkable as the book is elsewhere singularly free from misprints. It seems unfortunate, too, that though an imposing list of incompatibles is given, some of the more important are left to be gleaned from the articles on individual drugs. The hypodermic dose of apomorphia is given as one-eighth of a grain, which is rather large for ordinary use. The book is full of information, but the present edition will be thought by most readers in this country to contain either too much or too little for their purposes, since so many unimportant drugs are included and so many valuable ones omitted.

**Transactions of the Ophthalmological Society of the United Kingdom.** Vol. XX. London: J. & A. Churchill. 1900.—"The Ophthalmological Society of the United Kingdom" find, apparently, no difficulty in selecting from the interesting papers read during the session sufficient material to make an excellent volume, and in the "Transactions of their 19th Session" they present to the medical world a book well worthy of an extensive perusal. We are always struck at once with the beauty of the illustrations which are contained in this book, both of the fundus and external parts of the eye: their clearness and definition point to a great amount of care in their production. The Bowman lecture for the year is always included in these

*Transactions*, and the lecture in this number was ably given by Mr. R. Marcus Gunn, who chose as his subject "Visual Sensation," and discussed it in a most exhaustive manner. There are many other interesting papers and instructive short notes on various matters connected with this specialty, and we can confidently recommend the book to any practitioner who takes an interest in, and has a little time to devote to, the subject.

**Annual Report of the Smithsonian Institution, 1896.** Washington: Government Printing Office. 1898.—In the above report there are several articles to which the attention of readers of this *Journal* may be drawn. It must suffice, however, to indicate their titles and authors. The first is a discussion of the "Efficiency of the Animal as a Prime Mover," by R. H. Thurston, who reaches the conclusion that the vital machine must operate through methods of energy transformation as yet unknown to science. The second is Sir Michael Foster's Huxley Lecture on "Recent Advances in Science, and their Bearing on Medicine and Surgery." This is followed by Sir J. Burdon-Sanderson's Royal Institution Lecture on "Ludwig and Modern Physiology." Less well known to English readers is Dr. Simon Henry Gage's presidential address to the American Microscopical Society on "The Processes of Life Revealed by the Microscope: a Plea for Physiological Histology." Sir John Murray's paper read before the third International Zoological Congress on the "General Conditions of Existence and Distribution of Marine Organisms," and Dr. Heim's paper on the "Biologic Relations between Plants and Ants" will appeal to students of bionomics; while Dr. Theodore Gill's paper on "Some Questions on Nomenclature" will prove of interest to systematists. In his presidential address on "The War with the Microbes," Dr. E. A. de Schweinitz deals with bacterial poisons. There are in addition to the above several interesting anthropological papers, especially one, admirably illustrated, entitled "Preliminary Account of an Expedition to the Pueblo Ruins near Winslow, Arizona."

**Transactions of the College of Physicians of Philadelphia.** Third Series. Vol. XXII. Philadelphia: [W. J. Dornan.] 1900.—This volume contains many papers of great interest. Particularly interesting is an account, by Dr. Packard, of medical societies in the United States founded before 1787, and of some old certificates of proficiency in medicine; whilst Dr. Weir Mitchell prints some manuscript letters of Jenner which are in the possession of the College, and are not in any of the various lives of Jenner. We notice that the placarding of houses for contagious disease which is carried out by the Sanitary Authority in some cities in the States seems not to be a success, and entails so much inconvenience on the inmates of the house that the law, where it is in force, is very often evaded. There are a number of good contributions; perhaps as they form a valuable group of papers on the same subject, we may specially call the attention of our readers to the articles by Dana on tic doloureux, and to those on the surgery of the Gasserian ganglion and fifth nerve by Drs. Keen, Spiller, Cushing, Davis, Dercum, and Abbe.

**Transactions of the Medical Society of the State of New York.** Published by the Society. 1901.—The President, Dr. A. M. Phelps, of New York, for his anniversary address, chose the subject of "Radical Cure of Inguinal Hernia," and he concludes his description as follows: "We have considered the subject of hernia from the beginning of the Christian era down to the beginning of this the twentieth century, and I believe that we have arrived at the last stage of perfection in the technique of the operation for its radical cure." Of the many other papers reported, it is difficult to select any for especial mention, as all of them show a degree of healthy vitality of which many other societies may well be envious.

**The Edinburgh Medical Journal.** New Series. Vol. VIII. Edinburgh: Young J. Pentland. 1900.—Of the numerous and original papers contained in this half-yearly volume, one of the most noteworthy at the present time is that by Dr. Vincent Dormer Harris on the feeding of phthisical patients in relation to the wasting of the body, in which he gives many important details on the two chief means at our disposal for the treatment of wasting in phthisis, even when there is marked fever present, by rest and regulated feeding.

**The Edinburgh Medical Journal.** New Series. Vol. IX. Edinburgh: Young J. Pentland. 1901.—This volume contains the reprint of the Morison lectures for 1900, delivered by Dr. G. W. Balfour. The subject chosen was "The Borderland," in which the ripe experience of the author could give many an interesting story. Dr. Horace C. Colman's analysis of eighty-seven cases of pernicious anæmia, with an inquiry into the after-history of twenty-two reported cures, is well worthy of study; as is also Dr. Frederick T. Roberts's rational and comprehensive study of the hepatic system.

**Transactions of the American Laryngological Association.** New York: Carey Printing Co. 1901.—The work of the American Laryngological Association is second to no other similar society in the world in point of quality, and a perusal of the *Transactions* for 1901 shows that the high standard of former years is well maintained. It is difficult to select any one paper for special praise, but we may allude to Bryson Delavan's masterly criticism on the statistics of the operations for malignant diseases of the larynx. Many interesting and rare cases are described by other writers in this volume.

**Transactions of the Clinical Society of London.** Vol. XXXIII. London: Longmans, Green, and Co. 1900.—As is usual in the transactions of this society, the papers which are brought together in this volume form a most valuable and interesting series of clinical studies, every one of which is well worth reading. One of the most suggestive is that by Dr. Caton, on the "Prevention of Valvular Disease in Acute Rheumatism," in which he pleads that "we should regard each case of acute rheumatism, not merely as *rheumatism*, but first and before everything else, as *a case of impending peril to the heart*." In his hands the treatment that he recommends seems to have been of singular efficacy in warding off this complication. Mr. Barker records twelve cases of perforating gastric ulcer treated by operation, and in his paper gives a most ingenious and probable explanation of the disappearance of the liver dulness after perforation of the stomach. Messrs. Jones and Tubby give a further series of cases of angular deformity from Pott's disease treated by manual rectification. One of the conclusions that they come to is that "so far from inducing paraplegia, the operation is actually a curative measure." The volume is beautifully illustrated with photographs and skiagrams.

**Transactions of the Medical Society of London.** Vol. XXIII. London: Harrison and Sons. 1900.—The high standard of the papers brought before the Medical Society of London is fully brought out by the excellence of the present volume, which contains Sir William Banks's exceedingly able "Practical Observations on Cancer of the Breast," Dr. Caley's "Prognosis in Appendicitis," and other papers of equal interest and importance. It is impossible to read the book without finding the most modern views on the varied subjects of which it treats.

**Medico-Chirurgical Transactions.** Vol. LXXXIII. London: Longmans, Green & Co. 1900.—The present volume contains a full report of the discussion on the open-air treatment of tuberculosis which took place at one of the Society's meetings, introduced by a paper by Dr. J. Kingston Fowler. It will well repay a careful perusal, giving as it does a good epitome of our present knowledge of the subject. Another contribution of importance is a valuable paper by Mr. Charles A. Ballance on "The Conduct of the Mastoid Operation for the Cure of Chronic Purulent Otorrhœa." This paper is illustrated by several plates, rendering it easy to follow the details of the operation. Much of the technique introduced by Mr. Ballance is quite new, and although it is perhaps somewhat more complicated than is absolutely necessary in most cases, yet judging from the author's published cases it seems to yield better results than have hitherto been obtained.

**Index-Catalogue of the Library of the Surgeon-General's Office, United States Army.** Second Series. Vol. V. Enamel—Fyuner. Washington: Government Printing Office. 1900.—The present volume of this invaluable work includes 6,825 author-titles, 7,645 subject-titles of separate books and pamphlets, and 40,045 titles of articles in periodicals. It is indispensable to any medical library.

